# A Novel Feruloyl Esterase-Producing Strain ZH-1 Belonging to *Bacillus altitudinis* from Baijiu Distiller’s Grains and Its Enzyme Cocktail with No Detectable β-Xylosidase Activity for Producing Functional Sugars from Arabinoxylan-Type Substrates

**DOI:** 10.3390/foods15203642

**Published:** 2026-10-14

**Authors:** Ruonan Wang, Yi Yang, Mengyang Huang, Xiaoge Li, Xinying Wu, Zihan Zhu, Jing Yang, Kongyang Wu, Zitai Sang

**Affiliations:** 1 College of Life Science, Luoyang Normal University, Luoyang 471934, Chinakywu2007@126.com (K.W.); sangzitai@lynu.edu.cn (Z.S.); 2 College of Forestry, Henan Agricultural University, Zhengzhou 450046, China; yangyi023@henau.edu.cn

**Keywords:** *Bacillus altitudinis*, feruloyl esterase, high-value compounds, enzyme cocktail, enzymatic hydrolysis, arabinoxylan, wheat bran

## Abstract

Arabinoxylan-rich agricultural residues are promising feedstocks for the production of high-value functional sugars and ferulic acid, yet their efficient enzymatic conversion is hindered by the complex crosslinked structures. This study aimed to isolate a novel feruloyl esterase-producing strain from Baijiu distiller’s grains and evaluate its enzyme cocktail for arabinoxylan degradation. A novel strain, *Bacillus altitudinis* ZH-1, was isolated and systematically characterized. Through one-factor-at-a-time optimization of both the medium and cultivation conditions, the feruloyl esterase activity was substantially increased (15.78-fold) compared with the activity obtained in the unoptimized Luria-Bertani (LB) medium. Genomic analysis revealed 103 unique carbohydrate-active enzyme (CAZyme)-encoding genes, including endoxylanases, α-L-arabinofuranosidases, and three putative feruloyl esterases. Studies on enzymatic properties revealed that the extracellular enzyme cocktail exhibited a hemicellulase-dominated profile and no detectable β-xylosidase activity under the assay conditions, with the three key enzymes showing over 76% of their maximum activity at pH 6.0 and 50 °C. In the degradation of insoluble wheat arabinoxylan, the enzyme cocktail released L-arabinose (69.00 mg/g substrate) and a xylooligosaccharides-equivalent fraction (244.00 mg/g substrate), which together accounted for 99.57% of total reducing sugars, along with ferulic acid (1.23 mg/g substrate). When applied to de-starched wheat bran, the enzyme cocktail achieved reducing sugar yields comparable to the commercial enzyme CTec2, while producing significantly higher levels of L-arabinose and xylooligosaccharides. These findings highlight the ZH-1 enzyme cocktail as an arabinoxylan-targeting biocatalyst with no detectable β-xylosidase activity under the assay conditions, showing promising potential for producing high-value functional sugars and ferulic acid from agricultural residues.

## 1. Introduction

Arabinoxylan (AX) is one of the major hemicellulosic polysaccharides in cereal cell walls and agricultural residues, such as wheat bran, corn bran, and other cereal crops [1]. As a dietary fiber, AX has been associated with multiple health benefits, including prebiotic effects, improved gut health, and modulation of blood glucose levels [2,3]. AX-rich agricultural residues can be used as inexpensive materials for the production of antioxidant ferulic acid (FA) and functional sugars such as prebiotic xylooligosaccharides (XOS) and the low-calorie sweetener L-arabinose, among others [4]. XOS acts as a well-known prebiotic that selectively stimulates the proliferation of beneficial gut microbiota [5]. L-arabinose, as a low-glycemic-index sweetener, helps suppress postprandial blood glucose elevation [6]. FA is one of the major phenolic acids abundant in plant cell walls, with major dietary sources including cereal brans, sugar beet pulp, and maize bran; its ester-bound content in arabinoxylan-rich plant biomass typically ranges from approximately 1 to 30 mg/g dry biomass [7,8]. It exhibits multiple health-promoting bioactivities, such as antioxidant, anti-inflammatory, and neuroprotective effects [8,9].

AX mainly consists of a β-1,4-xylan backbone with arabinose residues, which are partially esterified with FA. Notably, FA can further form crosslinks with lignin monomers, constituting a lignin-carbohydrate complex (LCC), which is a key factor contributing to the low enzymatic conversion efficiency of AX-rich agricultural residues [10,11]. Given the complex structure of AX, its efficient conversion to high-value-added products requires not only xylanases but also side-chain-cleaving enzymes, especially arabinofuranosidases and feruloyl esterases (FAE) [12]. FAE specifically hydrolyzes the ester linkages between ferulic acid and arabinose residues, disrupting the FA-mediated crosslinking within the LCC, thereby reducing substrate recalcitrance and improving enzyme accessibility [7]. To date, a wide range of microorganisms isolated from diverse sources have been reported to produce FAE, such as soil [13], silages [14], sheep rumen [15], and mouse feces [16]. However, as noted by Rahmani et al. [17] and confirmed by more recent studies [13,14], only a limited number of wild-type bacterial strains have been reported to produce feruloyl esterase activity above 1000 U/L in submerged fermentation. Thus, continuous screening for highly active FAE-producing strains to meet industrial demands is still necessary.

Distiller’s grains, a major solid by-product of Chinese Baijiu fermentation, are rich in hemicellulose (mainly arabinoxylan) with high levels of esterified ferulic acid [9,18]. Additionally, it has been reported that unique microbial communities with potent degradation capabilities exist in distiller’s grains [19]. In recent years, several FAE-producing microorganisms have been isolated from traditional Chinese fermentation systems (Table S1), including strains belonging to the genera *Acetobacter*, *Klebsiella*, and *Penicillium* [20,21,22]. In addition, several highly efficient FAE producers have been isolated from alternative sources, such as soil. Representative examples include *Bacillus pumilus* SK52.001 (195 U/L) [13] and *Aspergillus terreus* (6700 U/L) [23], which exhibited remarkable feruloyl esterase activities. These findings indicate that highly efficient FAE producers are widely distributed in diverse environmental niches. Considering that diverse microbial communities exist in distiller’s grains [19], distiller’s grains are expected to harbor rich FAE-producing microbial resources, making them a promising source for arabinoxylan-rich biomass valorization. However, the isolation of microorganisms with high feruloyl esterase activity from distiller’s grains and the optimization of fermentation processes for enzyme production remain largely unexplored.

In this study, to the best of our knowledge, we report the first isolation and characterization of a novel feruloyl esterase-producing *Bacillus altitudinis* ZH-1 from Baijiu distiller’s grains. We systematically optimized its fermentation conditions and performed whole-genome sequencing to decipher its genetic potential for lignocellulose degradation. The enzymatic properties of its extracellular enzyme cocktail were characterized, and its performance in hydrolyzing both model and natural arabinoxylan-rich substrates was evaluated, with a comparative analysis against the commercial enzyme CTec2. Our findings demonstrate that the ZH-1 extracellular enzyme cocktail, with no detectable β-xylosidase activity under the tested conditions, acts as a highly efficient and selective biocatalyst for the production of high-value functional sugars from agricultural residues, offering a promising new tool for wheat bran valorization.

## 2. Materials and Methods

### 2.1. Chemicals and Substrates

Ferulic acid (FA), ethyl ferulate (EFA), 4-nitrophenyl-β-D-glucopyranoside (pNPG), and 4-nitrophenyl-β-D-cellobioside (pNPC) were purchased from Yuanye Bio-Technology Co., Ltd. (Shanghai, China). Methyl ferulate (MFA) was purchased from Aladdin Biochemical Technology Co., Ltd. (Shanghai, China). Carboxymethyl cellulose sodium salt (CMC-Na), D-glucose, D-xylose, and L-arabinose were purchased from Tokyo Chemical Industry Co., Ltd. (TCI, Tokyo, Japan). Xylobiose (X2), xylotriose (X3), xylotetraose (X4), xylopentaose (X5), xylohexaose (X6), beechwood xylan (BWX), sugar beet arabinan (SBA), insoluble wheat arabinoxylan (I-WAX), 4-nitrophenyl-α-L-arabinofuranoside (pNPAf), and 4-nitrophenyl-β-D-xylopyranoside (pNPX) were purchased from Megazyme International (Wicklow, Ireland). 4-nitrophenol (pNP) was acquired from Macklin Biochemical Co., Ltd. (Shanghai, China). Commercial cellulase (Cellic^®^ CTec2) was obtained from Sigma-Aldrich (St. Louis, MO, USA). The Bradford protein assay reagent was purchased from Bio-Rad Laboratories (Hercules, CA, USA). Other unstated analytical grade chemicals and reagents were purchased from Sinopharm Chemical Reagent Co., Ltd. (Shanghai, China).

Wheat bran was collected from a local flour mill in Luoyang, China, and subsequently sieved through a series of screens to obtain fractions of untreated, 18-mesh, 50-mesh, and 70-mesh particle sizes. De-starched wheat bran (DSWB) was prepared according to a previously described procedure [24]. According to the literature data, raw wheat bran typically contains 30–40% arabinoxylan (AX) and 11–24% starch on a dry-weight basis [25]. After destarching, most starch is removed, which increases the relative proportion of AX in DSWB [4]. It should be noted that these are the general literature-reported compositional ranges for wheat bran; no batch-specific physicochemical measurement was carried out for the raw wheat bran or DSWB used in this work.

### 2.2. Isolation, Screening, and Identification of Feruloyl Esterase-Producing Strain

Distiller’s grains were collected from a strong-aroma (Nongxiangxing) Baijiu distillery in Hebin Street, Yichuan County, Luoyang, Henan Province, China, in March 2024. Samples were obtained from discarded spent grains after distillation at the end of the full fermentation cycle. Three independent samples were collected from three different locations within the outdoor spent grain pile using 50 mL sterile centrifuge tubes. Samples were transported to the laboratory on ice within 2 h and stored at 4 °C prior to strain isolation, which was performed within 24 h of sampling. A 10 g sample was suspended in 90 mL of sterile distilled water and shaken at 180 rpm and 37 °C for 30 min. After serial dilution, 100 μL aliquots were spread onto Luria–Bertani (LB) agar plates and incubated at 37 °C for 72 h. Single colonies were purified by repeated streaking on LB agar plates. The purified strains were maintained in LB broth containing 20% (*v*/*v*) glycerol at −80 °C for subsequent experiments. The screening LB plates containing EFA were prepared as described by Wu et al. [20], and the purified isolates were point-inoculated onto the plates and incubated at 37 °C for 72 h. Colonies producing clear halos were selected as feruloyl esterase-positive. Among these, one isolate, designated ZH-1, which produced the largest clear zones, was chosen for further experiments. The strain *B. altitudinis* ZH-1 has been deposited in the China Center for Type Culture Collection (CCTCC) under the accession number CCTCC M 20251468.

For taxonomic identification, the genomic DNA of strain ZH-1 was extracted using a commercial bacterial genomic DNA extraction kit (TIANGEN Biotech Co., Ltd., Beijing, China). The 16S rRNA gene was amplified by PCR and sequenced at Sangon Biotech Co., Ltd. (Shanghai, China). The resulting 16S rDNA sequence was compared with the NCBI (National Center for Biotechnology Information) database and the EzBioCloud database. A phylogenetic tree was constructed using the neighbor-joining method with MEGA 11, and the tree topology was refined and visualized using TVBOT (https://chiplot.online/tvbot.html; accessed on 11 June 2026).

### 2.3. Optimization of Fermentation Conditions for Feruloyl Esterase Production by Bacillus altitudinis ZH-1

*Bacillus altitudinis* ZH-1 preserved at −80 °C was activated on LB agar plates at 37 °C for 24 h. A single colony was then transferred into 50 mL of LB broth in a 150 mL flask and cultured at 37 °C with shaking at 150 rpm for 12 h to obtain a seed culture. The production medium was selected through preliminary screening (Table S2) and consisted of (per liter): glucose 30 g, yeast extract 60 g, wheat bran 30 g, KCl 0.05 g, MgSO_4_·7H_2_O 0.05 g, and K_2_HPO_4_ 0.10 g, with an initial pH of 6.5. All fermentations were conducted in 150 mL flasks containing 30 mL of medium. The seed culture (5%, *v*/*v*) was inoculated into LB medium (as the unoptimized control) and the production medium, respectively, and incubated at 37 °C with rotary shaking at 150 rpm for 72 h. After fermentation, the culture broth was centrifuged at 7200× *g* for 15 min, and the cell-free supernatant was collected as the crude enzyme for feruloyl esterase activity determination. Using methyl ferulate as substrate, feruloyl esterase activity was measured by high-performance liquid chromatography (HPLC) as described by Wang et al. [24]. Briefly, the reaction mixture contained 1 mM MFA in 50 mM citrate-Na_2_HPO_4_ buffer (pH 7.0) and 10 μL of crude enzyme in a total volume of 200 μL. The reaction was incubated at 50 °C for 10 min and terminated by adding 100 μL acetonitrile. The released ferulic acid was quantified by HPLC using a ferulic acid calibration standard curve (0–1 mM, R^2^ > 0.99). Blank reactions without enzyme or without substrate were included. Preliminary experiments confirmed that the enzyme amount and reaction time used were within the linear range of the assay. One unit (U) of enzyme activity was defined as the amount of enzyme that released 1 μmol of ferulic acid per minute under the assay conditions.

The effects of wheat bran particle size (untreated, 18-mesh, 50-mesh, or 70-mesh), initial medium pH (5.5–9.5), agitation speed (0, 100, 150, or 200 rpm; 0 rpm indicates static cultivation), incubation temperature (34 °C, 37 °C, or 40 °C), and incubation time (24–96 h) on FAE activity were investigated. Optimization was performed using a sequential one-factor-at-a-time approach, in which each subsequent parameter was optimized under the condition established in the preceding experiment.

### 2.4. Whole-Genome Sequencing and Bioinformatics Analysis of B. altitudinis ZH-1

The whole genome of *B. altitudinis* ZH-1 was sequenced by TSINGKE Biological Technology Co., Ltd. (Beijing, China) on the Oxford Nanopore platform. The filtered reads were assembled using Canu v1.5, and the resulting contigs were circularized with Circlator v1.5.5. Coding sequences (CDSs) were predicted using Prodigal v2.6.3, while tRNA and rRNA genes were identified via tRNAscan-SE v2.0 and Infernal v1.1.3, respectively. For functional annotation, the predicted protein sequences were aligned against the NCBI non-redundant (NR), protein family (Pfam), Swiss-Prot, TrEMBL (Translation of the EMBL database), KEGG (Kyoto Encyclopedia of Genes and Genomes), and eggNOG (evolutionary genealogy of genes: Non-supervised Orthologous Groups) databases using BLAST v2.2.29 (E-value < 1 × 10^−5^), and Gene Ontology (GO) terms were assigned using Blast2GO v2.5.

Carbohydrate-active enzymes (CAZymes) were initially annotated by the sequencing company through BLAST searches against the CAZy database. To verify the annotation, the protein sequences were further submitted to the dbCAN meta server (dbCAN3 server), which integrates HMMER, DIAMOND, and Hotpep searches against the CAZy family databases. A gene was counted as a CAZyme if it contained at least one canonical CAZyme domain. For genes encoding multi-domain proteins, the gene was counted once in the total gene number, while the individual domains were counted separately in the family-level distribution analysis. The verified CAZyme sequences were then subjected to BLASTP searches against the Protein Data Bank (PDB) database (E-value < 1 × 10^−5^) for functional prediction of CAZymes. Proteins with higher sequence similarity to those with resolved crystal structures were considered to share similar functions. The presence of signal peptides was predicted using SignalP v4.0, which suggested that several of the annotated proteins may be secreted.

The genome sequence data have been deposited in GenBank under the BioProject accession number PRJNA1476822, BioSample accession number SAMN60733002, and the Whole Genome Shotgun project accession number JBZBUO000000000. The version described in this paper is JBZBUO010000000. The genome sequence of strain ZH-1 was uploaded to the Type (Strain) Genome Server (TYGS, https://tygs.dsmz.de accessed on 30 September 2026) for whole-genome-based taxonomic analysis. The dDDH values were calculated using the recommended settings of Genome-to-Genome Distance Calculator (GGDC) 4.0 with formula d_0_. The OrthoANIu values were calculated using the EzBioCloud Average Nucleotide Identity (ANI) Calculator (https://www.ezbiocloud.net/tools/ani accessed on 30 September 2026). The reference genomes of *Bacillus altitudinis* 41KF2b^T^ (= DSM 21631^T^, GCA_000691145), *B. pumilus* ATCC 7061^T^ (GCA_000172815), and *B. safensis* subsp. *osmophilus* CECT 9344^T^ (= BC 9^T^, GCA_003254445) were retrieved from NCBI for comparison.

### 2.5. Preparation of the ZH-1 Extracellular Enzyme Cocktail and Its Enzymatic Characterization

The fermentation broth of strain ZH-1, cultivated under the optimal conditions (34 °C, static, 72 h), was centrifuged at 11,300× *g* and 4 °C for 15 min. The supernatant was dialyzed against deionized water at 4 °C for 24 h and lyophilized using a Beijing Sihuan LGJ-10C freeze dryer (Beijing Sihuan Scientific Instrument Co., Ltd., Beijing, China) at a condenser temperature of ≤−60 °C and a vacuum of <1 Pa for 120 h to obtain the ZH-1 extracellular enzyme cocktail powder, which was reconstituted in 20 mM Tris-HCl (pH 8.0) buffer prior to use in all subsequent activity and hydrolysis assays.

The specific activities of ZH-1 extracellular enzyme cocktail were assayed using the following substrates at the indicated concentrations: 0.5% (*w*/*v*) CMC-Na for endoglucanase (EG); 10 mM pNPC for cellobiohydrolase (CBH); 10 mM pNPG for β-glucosidase (βG); 1% (*w*/*v*) BWX for endoxylanase (XYL); 1% (*w*/*v*) SBA for arabinanase (ARA); 1 mM pNPAf for α-L-arabinofuranosidase (ABF); 1 mM pNPX for β-xylosidase (βX); and 1 mM MFA or EFA for feruloyl esterase (FAE). The enzyme amount and total reaction volume were as follows: 50 μL enzyme (0.1 mg/mL) in 200 μL total volume for CMC-Na, 50 μL enzyme (0.5 mg/mL) in 100 μL total volume for pNPC, 50 μL enzyme (0.1 mg/mL) in 100 μL total volume for pNPG, 100 μL enzyme (0.002 mg/mL) in 200 μL for BWX, 100 μL (0.1 mg/mL) enzyme in 200 μL for SBA, 10 μL (0.01 mg/mL) enzyme in 100 μL for pNPAf and pNPX, and 10 μL enzyme (0.5 mg/mL) in 200 μL for MFA or EFA. EG, XYL, and ARA activities were measured using the 3,5-dinitrosalicylic acid (DNS) method with absorbance read at 540 nm [4,26]. CBH, βG, ABF, and βX activities were assayed by measuring the release of p-nitrophenol (pNP) at 405 nm [4,26]. Feruloyl esterase activity was determined by HPLC at 320 nm, based on the quantification of ferulic acid released from MFA and EFA, respectively [24]. All the reaction mixtures were incubated at 50 °C and pH 7.0 (50 mM citrate-Na_2_HPO_4_ buffer). The reaction time was 10 min for all substrates except CMC-Na, which was incubated for 30 min. Glucose (0.1–0.45 mg/mL, R^2^ > 0.99), xylose (0.1–0.5 mg/mL, R^2^ > 0.99), arabinose (0.1–0.45 mg/mL, R^2^ > 0.99), pNP (0–0.65 mM, R^2^ > 0.99), and ferulic acid (0–1 mM, R^2^ > 0.99) standards were used for calibration. Blank reactions without enzyme or without substrate were included. The enzyme amount and reaction time used were confirmed to be within the linear range of each assay. Protein concentration was determined using the Bradford method with the Bio-Rad protein assay reagent, following the manufacturer’s instructions. One unit (U) of enzyme activity was defined as the amount of enzyme that released 1 μmol of the respective product per minute under the assay conditions.

The effects of temperature and pH on the activities of XYL, ABF, and FAE were measured at temperatures ranging from 30 to 80 °C (at 10 °C intervals) and over a pH range of 4.0–8.0 (in increments of 1.0 pH unit) using 50 mM citrate-Na_2_HPO_4_ buffer. The relative activity was calculated by defining the highest observed activity as 100%.

### 2.6. Application of the ZH-1 Extracellular Enzyme Cocktail in the Valorization of Arabinoxylan-Type Substrates

To evaluate the applied potential of the ZH-1 extracellular enzyme cocktail toward model and natural arabinoxylan-type substrates, degradation assays were conducted using insoluble wheat arabinoxylan (I-WAX) and de-starched wheat bran (DSWB). The reaction mixture contained 2% (*w*/*v*) substrate and 5 mg of total protein (based on Bradford assay) per gram of substrate in 50 mM citrate-Na_2_HPO_4_ buffer (pH 6.0) in a total volume of 0.5 mL, and was incubated at 40 °C with shaking at 200 rpm. For I-WAX hydrolysis, the reaction was carried out at both 40 °C and 50 °C. Individual reaction mixtures were prepared for each time point and stopped at 1 h and 12 h, respectively. For DSWB hydrolysis, the incubation was performed at 40 °C for 48 h and a parallel experiment was conducted under identical conditions using the commercial cellulase preparation Cellic^®^ CTec2 (Sigma-Aldrich, St. Louis, MO, USA) at the same protein loading (5 mg/g substrate), serving as a reference to the ZH-1 extracellular enzyme cocktail. Control reactions without enzyme addition were performed under identical conditions to distinguish enzymatic hydrolysis from non-enzymatic release. In these no-enzyme controls, no significant release of reducing sugars, monosaccharides (glucose, xylose, and arabinose), or ferulic acid was detected.

The reaction was terminated by boiling for 10 min after a brief centrifugation (16,260× *g*, 1 min). The inactivated samples were then centrifuged at 16,260× *g* for 5 min, and the supernatants were filtered through 0.22 μm filters. The resulting hydrolysates were analyzed for total reducing sugar content using the DNS method [26], for ferulic acid release by HPLC [24], for monosaccharide (glucose, xylose, and arabinose) release by HPLC (Agilent 1290, Agilent Technologies, USA), and for oligosaccharide composition by thin-layer chromatography (TLC) following previously established protocols [27]. Glucose, xylose, arabinose, and ferulic acid standards were used for calibration of the DNS and HPLC analyses. All hydrolysis data were corrected by subtracting the values obtained from the corresponding no-enzyme controls.

### 2.7. Statistical Analysis

All experiments were performed with three independent biological replicates (*n* = 3), defined as either independent fermentation batches or independent hydrolysis reactions, and results are expressed as mean ± standard deviation (SD). No additional analytical (technical) replicate measurements were performed; each biological replicate sample was measured once. Statistical analyses were conducted using GraphPad Prism 9.5.1 (GraphPad Software, La Jolla, CA, USA). For comparisons between two groups, an unpaired two-tailed *t*-test with Welch’s correction was applied. For multiple comparisons involving one independent variable, one-way analysis of variance (ANOVA) followed by Tukey’s post hoc test was used. For data involving two independent variables, two-way ANOVA followed by Tukey’s post hoc test was used to evaluate the main effects and their interaction. A *p*-value of < 0.05 was considered statistically significant.

## 3. Results and Discussion

### 3.1. Isolation and Identification of Feruloyl Esterase-Producing Strain ZH-1

**Figure 1:**
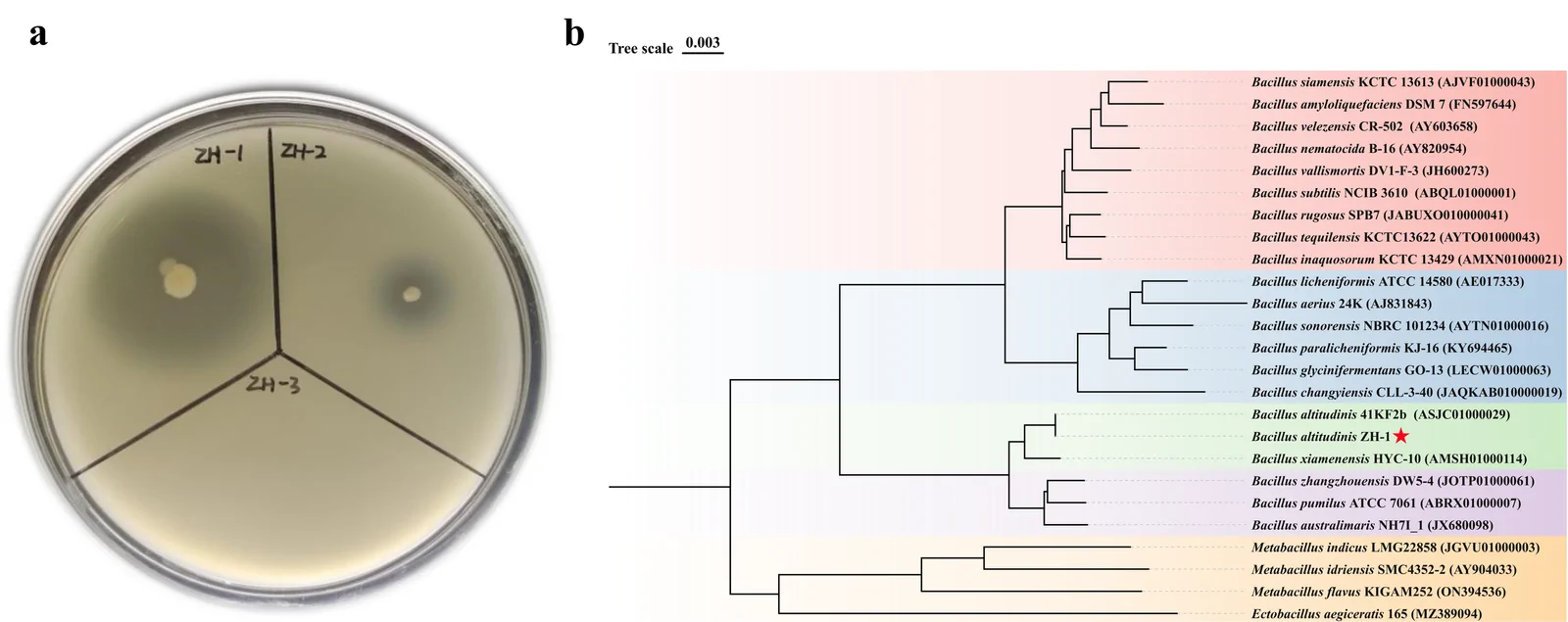
Isolation and identification of *B. altitudinis* ZH-1. (**a**) Hydrolysis zones formed by candidate strains on ethyl ferulate (EFA)-containing screening plates. (**b**) 16S rRNA gene phylogenetic tree of *B. altitudinis* ZH-1 with other species.

A total of 50 bacterial isolates were obtained from Baijiu distiller’s grain samples. Among them, two strains formed clear halo zones around the colonies on EFA-containing LB agar plates (Figure 1a), indicating the secretion of extracellular feruloyl esterases. At 72 h of incubation, strain ZH-1 exhibited a clear zone diameter (D) of 3.6 cm and a colony diameter (d) of 0.6 cm, yielding an enzymatic activity index (EI = D/d) of 6.0, while strain ZH-2 showed an EI of 3.5. According to the criterion that an EI ≥ 2.0 indicates a potential enzyme-producing microorganism [22], both strains were considered positive, with ZH-1 showing significantly higher activity. For comparison, the FAE-producing strain *Penicillium rubens* NRRL-1951 was reported to reach an EI of approximately 5.5 after 12 days of cultivation [28], while seven FAE-producing fungal strains isolated from the Amazon rainforest exhibited EI values ranging from 1.91 to 3.83 after 96 h [29]. Additionally, the FAE-producing strain *Penicillium oxalicum* M1816, isolated from wheat bran Qu, showed an EI of nearly 3.0 after 72 h of cultivation [22]. These results indicate that the EI of strain ZH-1 (6.0 within 72 h) is considerably higher than those of previously reported strains, further demonstrating its great feruloyl esterase-producing potential.

Using the genomic DNA of strain ZH-1 as a template, the 16S rRNA gene was amplified with universal primers 27F and 1492R and sequenced. BLAST analysis against the EzBioCloud 16S database revealed 100.00% identity of strain ZH-1 to the type strain *Bacillus altitudinis* 41KF2b^T^, confirming its identification as *B. altitudinis*, which was further supported by its clustering with *B. altitudinis* in the phylogenetic tree (Figure 1b). To further verify the taxonomic assignment of ZH-1, whole-genome-based comparisons were performed. The digital DNA-DNA hybridization (dDDH) analysis showed that strain ZH-1 shared the highest dDDH value of 95.40% with *Bacillus altitudinis* 41KF2b^T^ (= DSM 21631^T^), followed by *B. pumilus* ATCC 7061^T^ (85.80%) and *B. safensis* subsp. *osmophilus* CECT 9344^T^ (82.50%) (Table S3). Consistently, the OrthoANIu values between ZH-1 and these three type strains were 98.50%, 88.95%, and 89.04%, respectively. Both the dDDH (>70%) and OrthoANIu (≥95–96%) values exceeded the species delineation thresholds when compared with *B. altitudinis* 41KF2b^T^, whereas the values obtained for the other two strains fell below these thresholds. The G + C content difference between ZH-1 and *B. altitudinis* 41KF2b^T^ was only 0.12%. These results are consistent with the established species delineation thresholds for prokaryotes and strongly support the classification of strain ZH-1 as *Bacillus altitudinis*. Feruloyl esterase-producing microorganisms have been widely reported, including fungi, actinomycetes, and bacteria [8]. However, studies on such microorganisms derived from the Baijiu fermentation system remain limited, with only a few strains reported from Daqu and Jiupei [20,21]. Notably, *Bacillus altitudinis* is recognized as a versatile enzyme producer, with strains isolated from various environments being reported to produce esterases [30], xylanases [31,32,33], and β-glucosidase [34], demonstrating its potential in lignocellulose degradation and industrial biocatalysis. Despite this enzymatic diversity, feruloyl esterase production has rarely been documented in *Bacillus altitudinis*. Therefore, to the best of our knowledge, strain ZH-1 represents one of the first reports of a feruloyl esterase-producing *B. altitudinis* from Baijiu distiller’s grains, expanding the diversity of microbial resources for feruloyl esterase production.

### 3.2. Optimization of Fermentation Conditions for Feruloyl Esterase Production by B. altitudinis ZH-1

**Figure 2:**
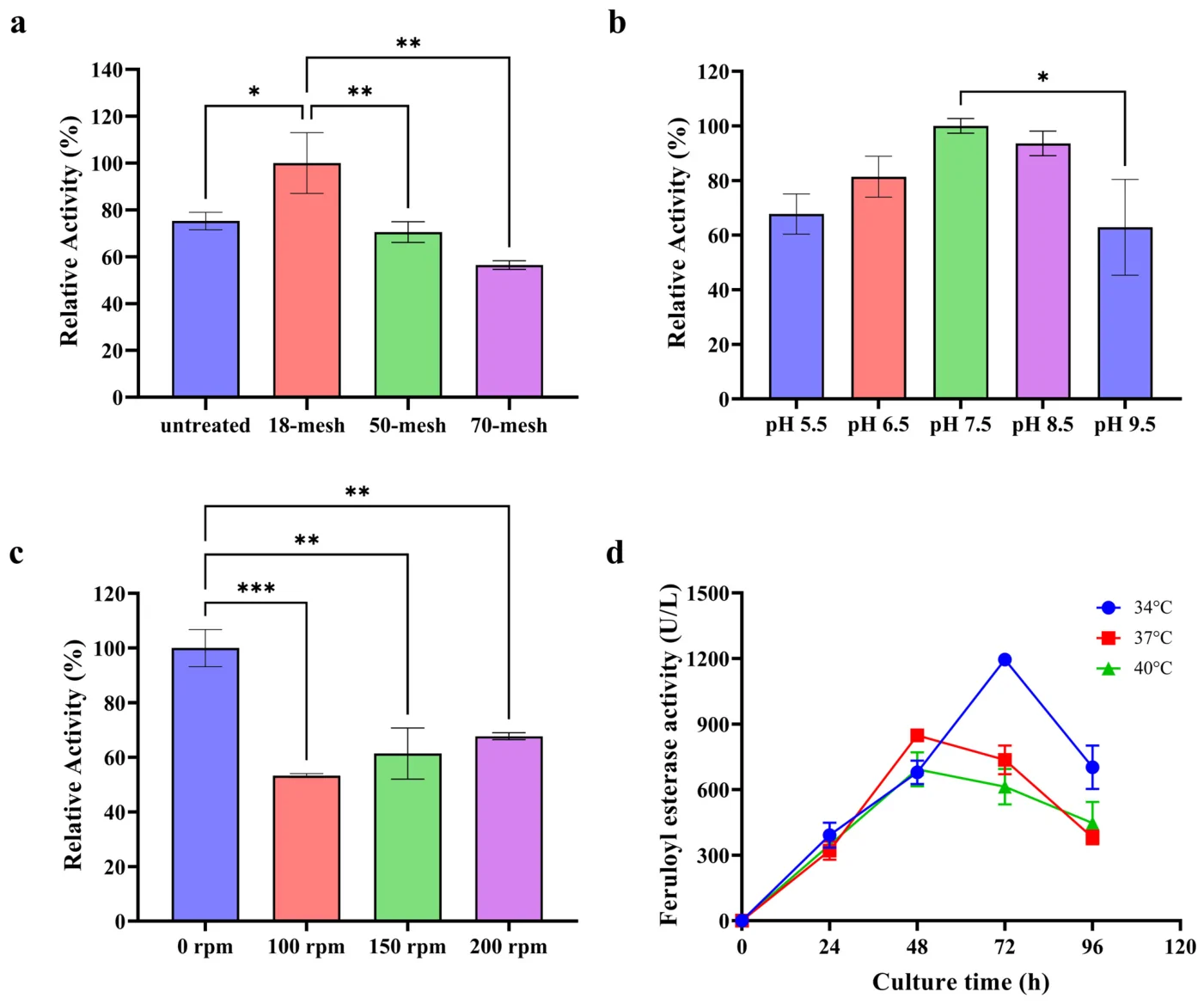
Effects of fermentation conditions on feruloyl esterase activity of *B. altitudinis* ZH-1. (**a**) Wheat bran particle size. (**b**) Initial pH. (**c**) Agitation speed. (**d**) Cultivation temperature and time. Data in (**a**–**c**) are presented as relative activity (%, with the highest value set as 100%), while data in (**d**) are shown as absolute enzyme activity (U/L). All data are presented as mean ± standard deviation (SD) (*n* = 3). Statistical significance is indicated by * (*p* < 0.05), ** (*p* < 0.01), and *** (*p* < 0.001) based on one-way analysis of variance (ANOVA) followed by Tukey’s post hoc test.

Extracellular enzyme production by microorganisms is known to be influenced by multiple factors, such as medium composition, pH, temperature, and cultivation time [35,36]. To determine the optimal fermentation conditions for strain ZH-1, the culture medium was first screened. The optimal medium composition was determined as follows: 30 g/L glucose, 60 g/L yeast extract, 30 g/L wheat bran, 0.05 g/L KCl, 0.05 g/L MgSO_4_·7H_2_O, and 0.10 g/L K_2_HPO_4_ (Table S2). Based on this medium, a one-factor-at-a-time sequential optimization strategy was employed to investigate the effects of wheat bran particle size, initial pH, agitation speed, and cultivation temperature and time on feruloyl esterase activity (Figure 2).

FAE is an inducible enzyme stimulated by complex carbon sources rich in esterified ferulic acid, such as wheat bran, for which particle size is a critical factor affecting microbial growth and enzyme production [23]. Wheat bran particle size significantly influenced enzyme production by strain ZH-1 (Figure 2a). The 18-mesh fraction yielded the highest activity, which was 1.33-fold higher than that obtained with untreated wheat bran. When the particle size was further reduced to 50-mesh and 70-mesh, the enzyme activities decreased significantly to 70.49% and 56.45% of that of the 18-mesh treatment, respectively. This indicated that excessively large particles may reduce substrate accessibility, whereas overly fine particles may increase viscosity and impede mass transfer and oxygen supply, thereby affecting enzyme production [23]. Therefore, 18-mesh wheat bran was selected for subsequent experiments. As shown in Figure 2b, strain ZH-1 demonstrated a remarkably broad pH adaptability for feruloyl esterase production. The maximum activity was achieved at pH 7.5, and high-level production was maintained across pH 6.5–8.5, whereas increasing the pH to 9.5 led to a significant decrease to 62.84% of the maximum. Agitation speed is also a major environmental parameter determining microbial growth [37]. As shown in Figure 2c, static cultivation (0 rpm) resulted in significantly higher enzyme activity compared with shaking conditions; at 100, 150, and 200 rpm, the activities were only 53.29%, 61.38%, and 67.71% of that under static conditions, respectively. This phenomenon may be attributed to the isolation of strain ZH-1 from Baijiu distiller’s grains, a solid-state fermentation ecosystem where microorganisms naturally grow under static or micro-aerobic conditions.

Based on the above results, the optimal conditions (18-mesh wheat bran, pH 7.5, and static cultivation) were used for further temperature and time optimization (Figure 2d). At all three temperatures tested, the enzyme activity first increased and then decreased. The maximum activity (1194.79 U/L) was observed at 34 °C after 72 h of cultivation, representing a 15.78-fold increase relative to the activity obtained in the unoptimized LB medium (75.70 U/L) under the initial cultivation conditions (37 °C, 150 rpm, 72 h). This enhancement was obtained through sequential screening of different medium types (Table S2), followed by one-factor-at-a-time optimization of wheat-bran particle size, initial pH, agitation speed, incubation temperature, and cultivation time. Peaks at 37 °C and 40 °C occurred earlier (48 h) but with lower values (848.04 and 692.97 U/L, respectively). At 72 h, the activities at 37 °C and 40 °C were 64.68% and 51.32% of that at 34 °C, respectively. Therefore, 34 °C and 72 h were determined as the optimal temperature and cultivation time.

To evaluate the FAE-producing potential of *B. altitudinis* ZH-1, its enzyme activity was compared with that of previously reported FAE-producing strains under submerged fermentation conditions (Table 1). Under the optimized conditions, strain ZH-1 produced 1194.79 U/L of FAE, which was considerably higher than most bacterial FAE producers, including *Bacillus pumilus* SK52.001 (195 U/L) [13], *Lactiplantibacillus pentosus* (7.11 U/L) [14], and *Klebsiella oxytoca* Z28 (35 U/L) [21]. Notably, the activity of ZH-1 also exceeded that of several fungal strains, such as *Humicola grisea* var. *thermoidea* (470 U/L) [37] and *Penicillium brasilianum* IBT-20888 (225 U/L) [38]. Among all the strains compared, only *Aspergillus terreus* showed a higher FAE activity (6700 U/L) than ZH-1, although it required a substantially longer fermentation time (120 h) [23]. These results indicate that strain ZH-1 is one of the most efficient bacterial FAE producers reported to date, achieving high enzyme activity within a relatively short fermentation period. The high FAE activity achieved under optimized conditions supports the potential of strain ZH-1 for cost-effective industrial enzyme production, which is relevant for the valorization of low-cost agricultural by-products such as wheat bran.

### 3.3. Genomic Features and CAZyme Annotation of B. altitudinis ZH-1

To gain insights into the genetic basis of feruloyl esterase production and the lignocellulolytic potential of *B. altitudinis* ZH-1, its complete genome was sequenced. The genome consists of a single circular chromosome of 3,732,220 bp with a G + C content of 41.39% (Figure S1). A total of 3759 protein-coding genes were predicted, with an average length of 872 bp, covering 87.86% of the genome. In addition, 81 tRNA and 24 rRNA genes were identified. Furthermore, functional annotation revealed that 2962, 2932, and 2723 genes were annotated in the eggNOG, GO, and KEGG databases, representing 78.80%, 78.00%, and 72.44% of the 3759 predicted genes, respectively (Table S4).

**Figure 3:**
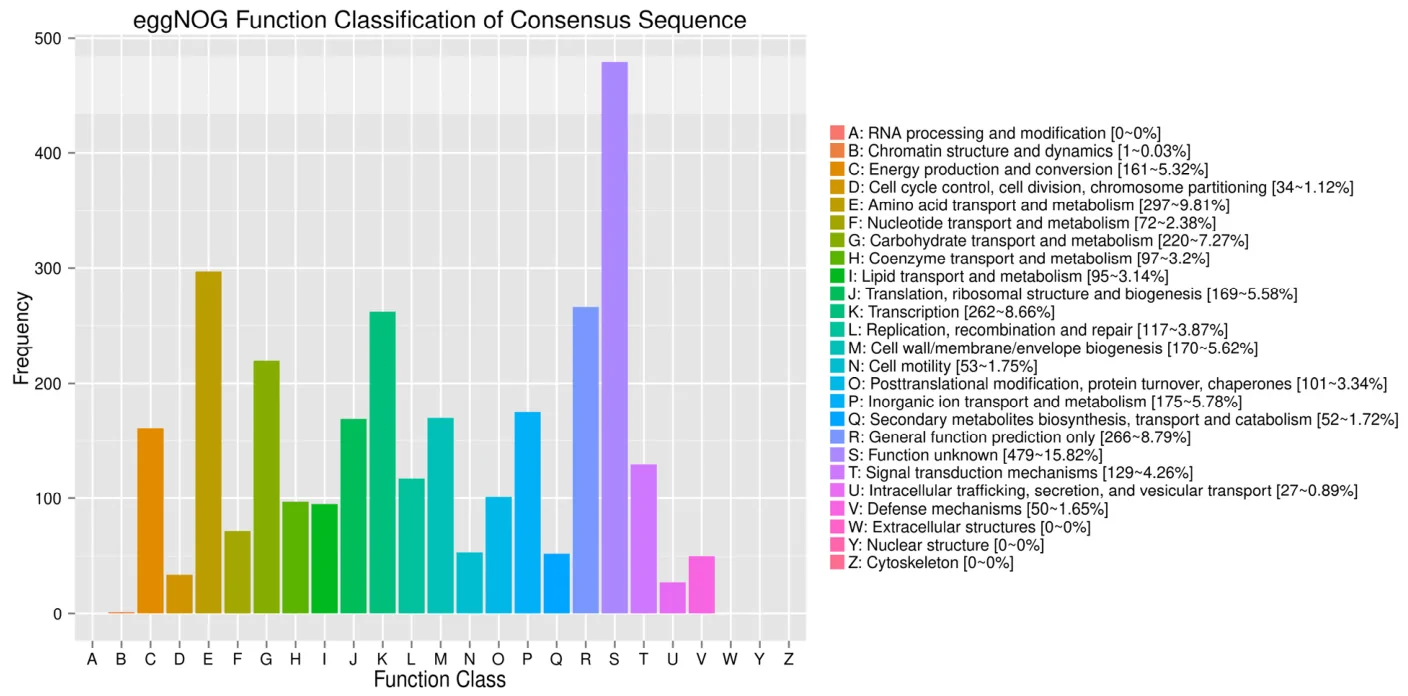
eggNOG functional classification of the *B. altitudinis* ZH-1 genome.

**Figure 4:**
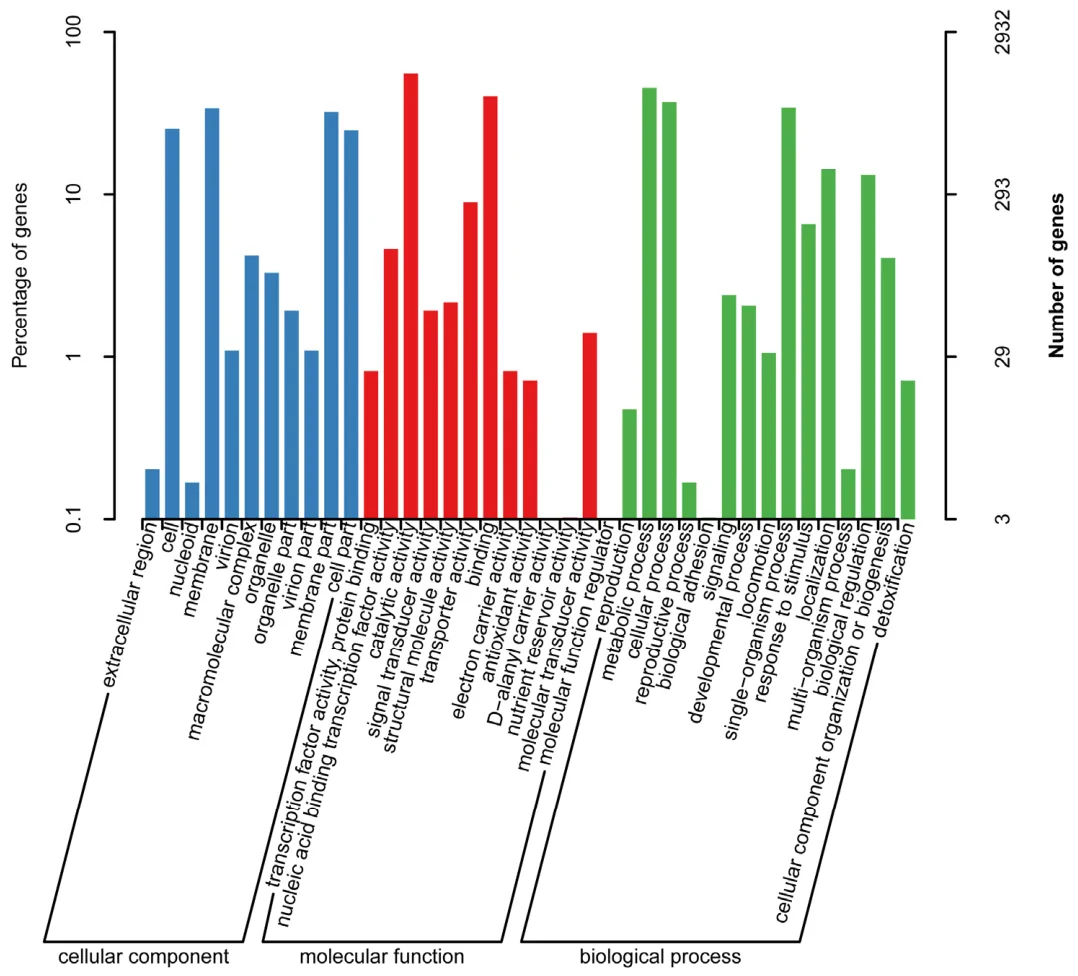
Gene Ontology (GO) functional classification of the *B. altitudinis* ZH-1 genome.

**Figure 5:**
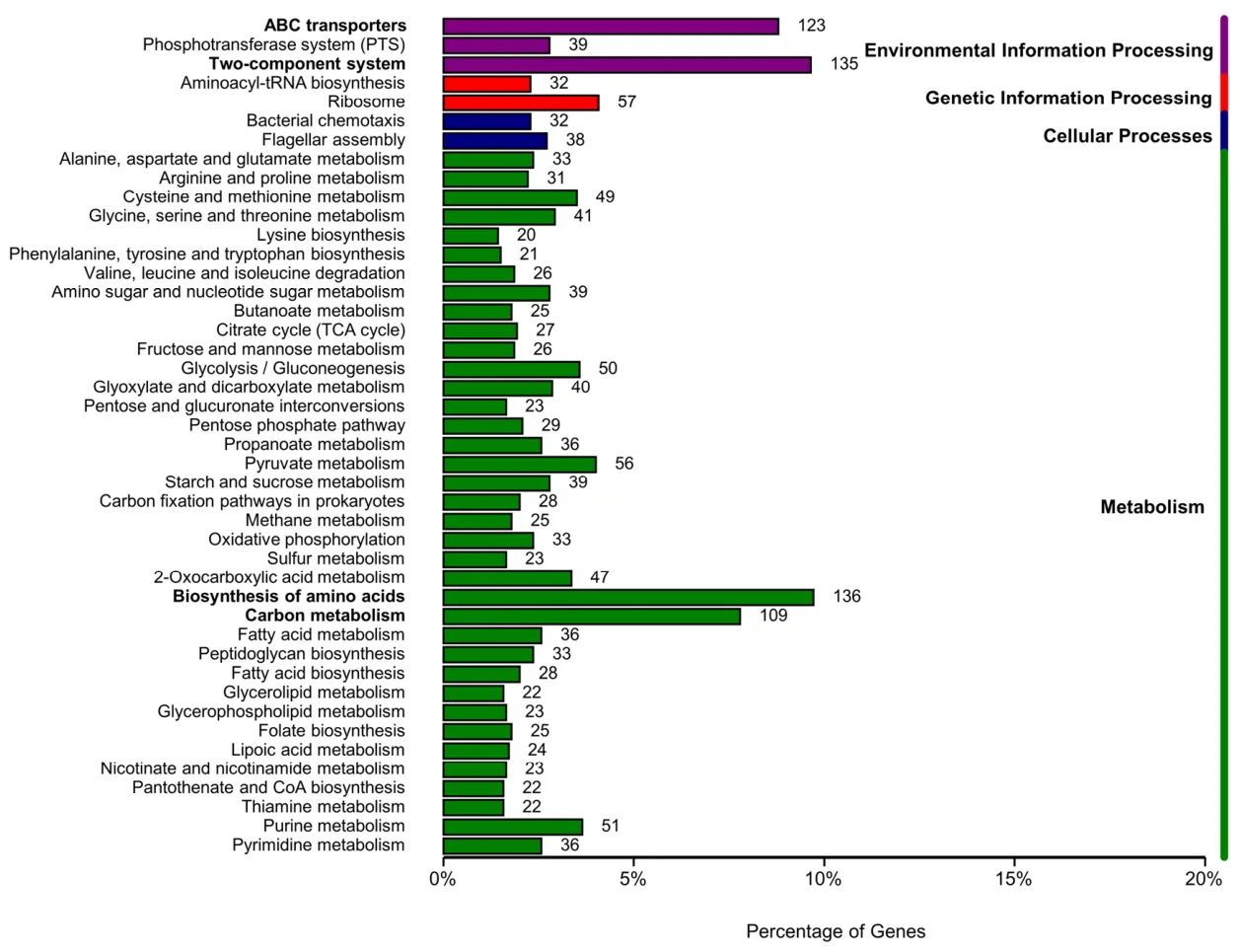
Kyoto Encyclopedia of Genes and Genomes (KEGG) pathway annotation of the *B. altitudinis* ZH-1 genome at the second level.

In the eggNOG database, the annotated genes were assigned to 21 functional categories (Figure 3). The most predominant categories were Function unknown (15.82%), Amino acid transport and metabolism (9.81%), General function prediction only (8.79%), Transcription (8.66%), and Carbohydrate transport and metabolism (7.27%). GO annotation revealed that the most prevalent terms were catalytic activity (15.75%), metabolic process (12.87%), binding (11.41%), and cellular process (10.53%), which collectively accounted for 60.30% of all GO annotations and were mainly assigned to the molecular function and biological process categories (Figure 4). KEGG pathway analysis showed that the most abundantly represented pathways were Biosynthesis of amino acids (9.44%), Two-component system (9.38%), ABC transporters (8.54%), and Carbon metabolism (7.57%) (Figure 5).

**Figure 6:**
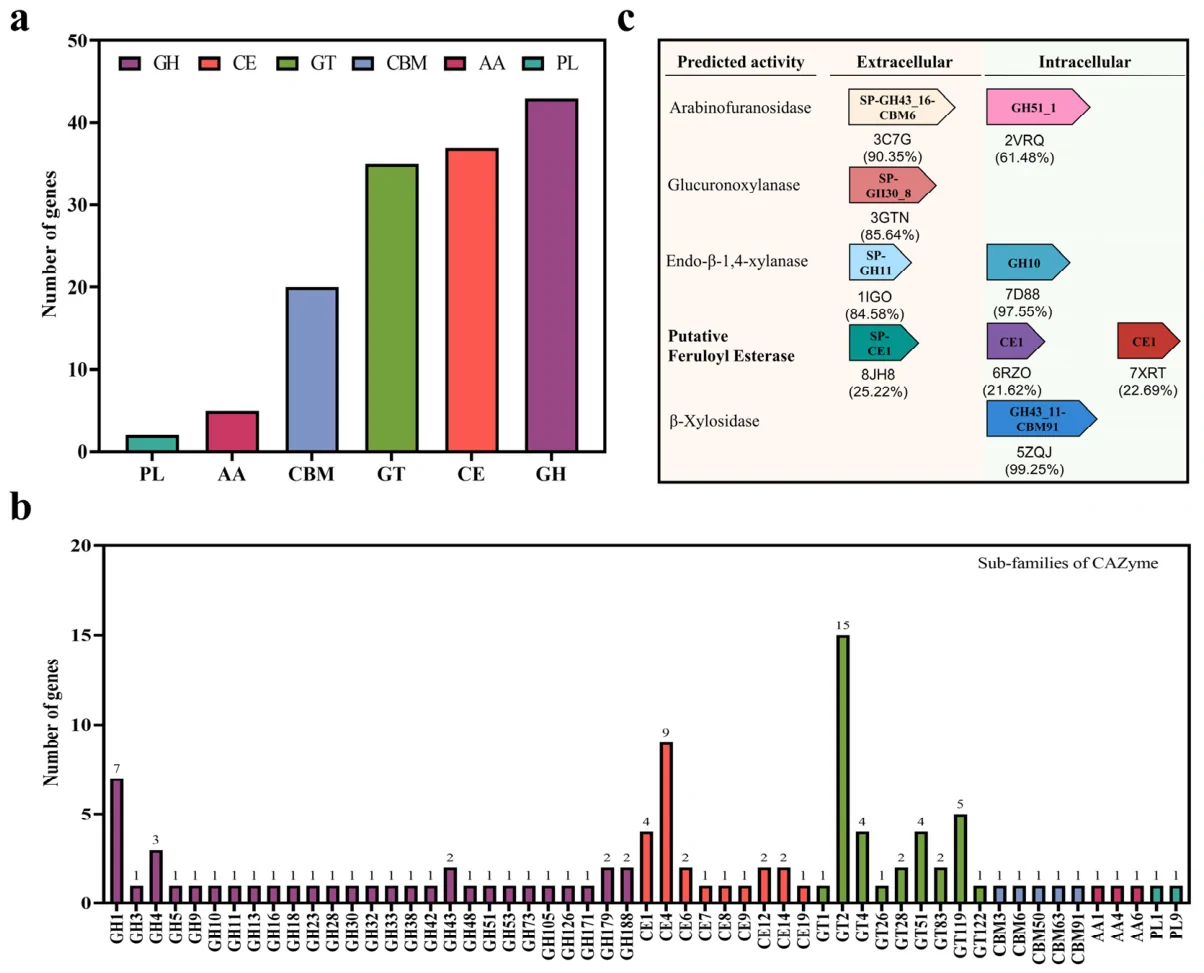
Carbohydrate-active enzyme (CAZyme) annotation of *B. altitudinis* ZH-1. (**a**) Distribution of CAZyme family assignments (domain counts) across six major functional classes. (**b**) Subfamily distribution of CAZymes. (**c**) Domain architecture and PDB-based homology analysis of key CAZymes. Colored blocks indicate functional domains (GH: glycoside hydrolase; CE: carbohydrate esterase; CBM: carbohydrate-binding module). SP denotes the presence of a signal peptide. The terms “extracellular” and “intracellular” indicate the presence or absence of a predicted signal peptide, respectively, and do not represent experimentally determined subcellular localization. The first line below each domain architecture shows the PDB ID of the most similar protein, and the second line indicates the sequence identity (%). The three CE1 genes were designated as putative feruloyl esterase-encoding genes based on low sequence identities (21.62–25.22%), and their actual FAE activity requires further biochemical validation.

To decipher the gene repertoire encoding lignocellulolytic enzymes in strain ZH-1, CAZy annotation was performed. CAZy annotation identified 106 CAZyme family assignments across six major categories (Figure 6a), with glycoside hydrolases (GHs, 38), glycosyl transferases (GTs, 35), carbohydrate esterases (CEs, 23), carbohydrate-binding modules (CBMs, 5), auxiliary activities (AAs, 3), and polysaccharide lyases (PLs, 2). After removing multi-domain redundancy, 103 unique CAZyme-encoding genes were identified. This number is comparable to those of other reported *B. altitudinis* strains, including SRL571 (101 genes), JYY-02 (105 genes), B12 (100 genes), and W3 (100 genes) (Table S5). Among these, *B. altitudinis* JYY-02, isolated from pine tree soil, exhibits β-glucosidase activity [34]. *B. altitudinis* SRL571, a halotolerant endophytic bacterium isolated from the inner leaf tissues of *Cakile maritima*, has been reported to utilize glucuronoxylan as its sole carbon and energy source by secreting two endoxylanases [43]. *B. altitudinis* W3 (previously named *B. pumilus*) isolated from raw gallnut honey has been reported to exhibit high CotA-laccase activity [44]. These findings indicate that *B. altitudinis* strains harbor diverse CAZyme repertoires tailored to their specific ecological niches.

The domains encoded by those 103 genes were further classified into 55 subfamilies (Figure 6b). The 38 GHs were distributed across 27 subfamilies, 23 CEs across 9 subfamilies, and 3 AAs across 3 subfamilies. To gain deeper insight into their potential functions, PDB-based homology analysis was performed to predict the key CAZymes potentially involved in cellulose and hemicellulose degradation in ZH-1, along with their putative functions (Table S6). The predicted cellulolytic enzymes include endo-β-1,4-glucanase and cellobiohydrolase; the hemicellulolytic enzymes encompass multiple endoxylanases, α-L-arabinofuranosidases, β-xylosidase, acetyl xylan esterases, and putative feruloyl esterases. In addition, a gene encoding mannanase was also predicted in the ZH-1 genome.

Signal peptide prediction further indicated that most hemicellulose-degrading enzymes contain signal peptides, suggesting that these enzymes may be secreted and may act on arabinoxylan (Figure 6c). Notably, among the CE1 members of ZH-1, three genes showed sequence identities of 21.62%, 22.69%, and 25.22%, with characterized feruloyl esterases, and alignment coverages of 71%, 70%, and 24%, respectively. Because of the low sequence identities (21.62–25.22%), these genes can only be regarded as putative feruloyl esterase-encoding genes, and their actual FAE activity requires further biochemical validation. One of them (GE003466) was predicted to contain a signal peptide (Figure 6c), suggesting its potential for secretion. The clear-zone formation on EFA-containing plates provides functional evidence for extracellular FAE activity in ZH-1, although the identity of the responsible gene remains to be confirmed.

### 3.4. Enzymatic Properties of the Extracellular Enzyme Cocktail of B. altitudinis ZH-1

**Figure 7:**
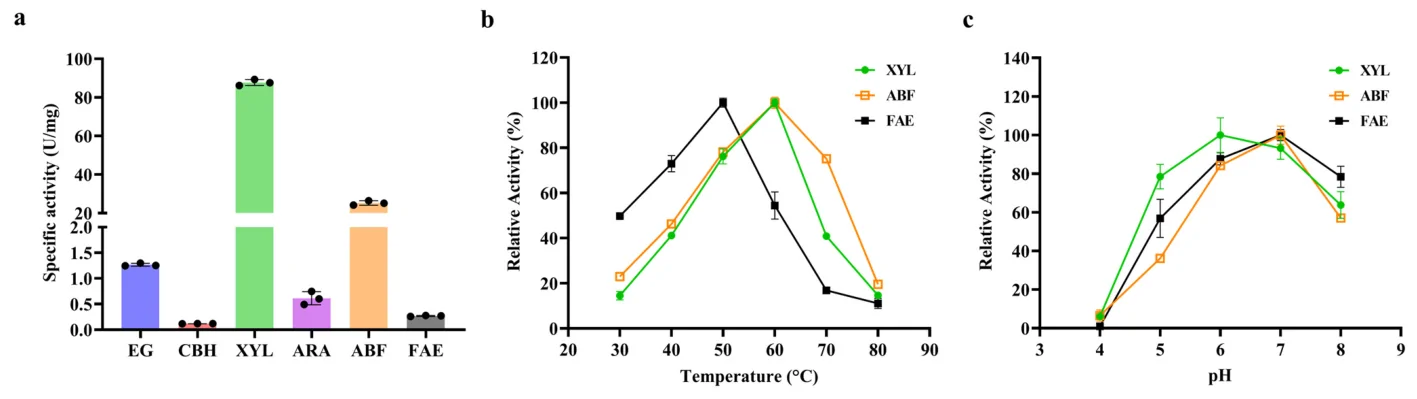
Characterization of the extracellular enzyme cocktail of *B. altitudinis* ZH-1. (**a**) The substrate specificity. (**b**) Effects of temperature on enzyme activity. (**c**) Effects of pH on enzyme activity. EG: endoglucanase; CBH: cellobiohydrolase; XYL: endoxylanase; ARA: arabinanase; ABF: α-L-arabinofuranosidase; FAE: feruloyl esterase. Data are presented as mean ± SD (*n* = 3).

Substrate specificity assays (Figure 7a) revealed that the ZH-1 extracellular enzyme cocktail collected under optimal fermentation conditions exhibited the highest specific activities toward beechwood xylan (BWX, 87.70 U/mg) and pNPAf (25.22 U/mg), indicating a predominance of endoxylanase (XYL) and α-L-arabinofuranosidase (ABF) activities. Among the hemicellulolytic activities, the cocktail showed moderate arabinanase (0.61 U/mg) and feruloyl esterase (0.27 U/mg) activities. In contrast, relative to the dominant endoxylanase activity, the cellulolytic activities were negligible (1.27 U/mg toward CMC-Na and 0.12 U/mg toward pNPC, corresponding to 1.4% and 0.14% of the endoxylanase activity, respectively). No activity was detected toward pNPX or pNPG under our assay conditions, indicating that β-xylosidase and β-glucosidase activities were absent from the extracellular enzyme cocktail prepared in this study. Collectively, these results indicate that the ZH-1 extracellular enzyme cocktail exhibits a hemicellulase-dominated profile and specifically targets arabinoxylan-type substrates, consistent with the CAZyme annotation results.

The optimal reaction temperatures and pH of XYL, ABF, and FAE were determined (Figure 7b,c). XYL and ABF showed optimal temperatures at 60 °C, while FAE exhibited a lower optimum of 50 °C. XYL and ABF remained active over 50–70 °C (>76% at 50 °C; >40% at 70 °C), whereas FAE activity declined sharply above 50 °C (54% at 60 °C). The optimal pH values were 6.0 for XYL and 7.0 for both ABF and FAE. FAE showed high activity at pH 6.0–8.0 (>78%) but decreased to 56% at pH 5.0, indicating a preference for neutral conditions. Notably, all three enzymes showed over 76% of their maximum activity at pH 6.0 and 50 °C, indicating that these conditions are compatible with the activities of all three enzymes. It should be noted that only initial pH- and temperature-dependent enzyme activities were characterized in this study; comprehensive enzymatic stability, including thermal stability and prolonged residual activity, was not evaluated. The optimal temperature and pH of XYL in this study (60 °C, pH 6.0) are consistent with those reported for other *B. altitudinis* strains, such as X-10 (45 °C, pH 6.0) [31], XYL17 (55 °C, pH 6.5) [32], and RS3025 (60 °C, pH 6.0) [45]. Moreover, these strains have demonstrated potential in agro-waste valorization, further supporting the lignocellulolytic ability of ZH-1.

### 3.5. Application of the Extracellular Enzyme Cocktail of B. altitudinis ZH-1 in Arabinoxylan-Type Substrate Hydrolysis

**Figure 8:**
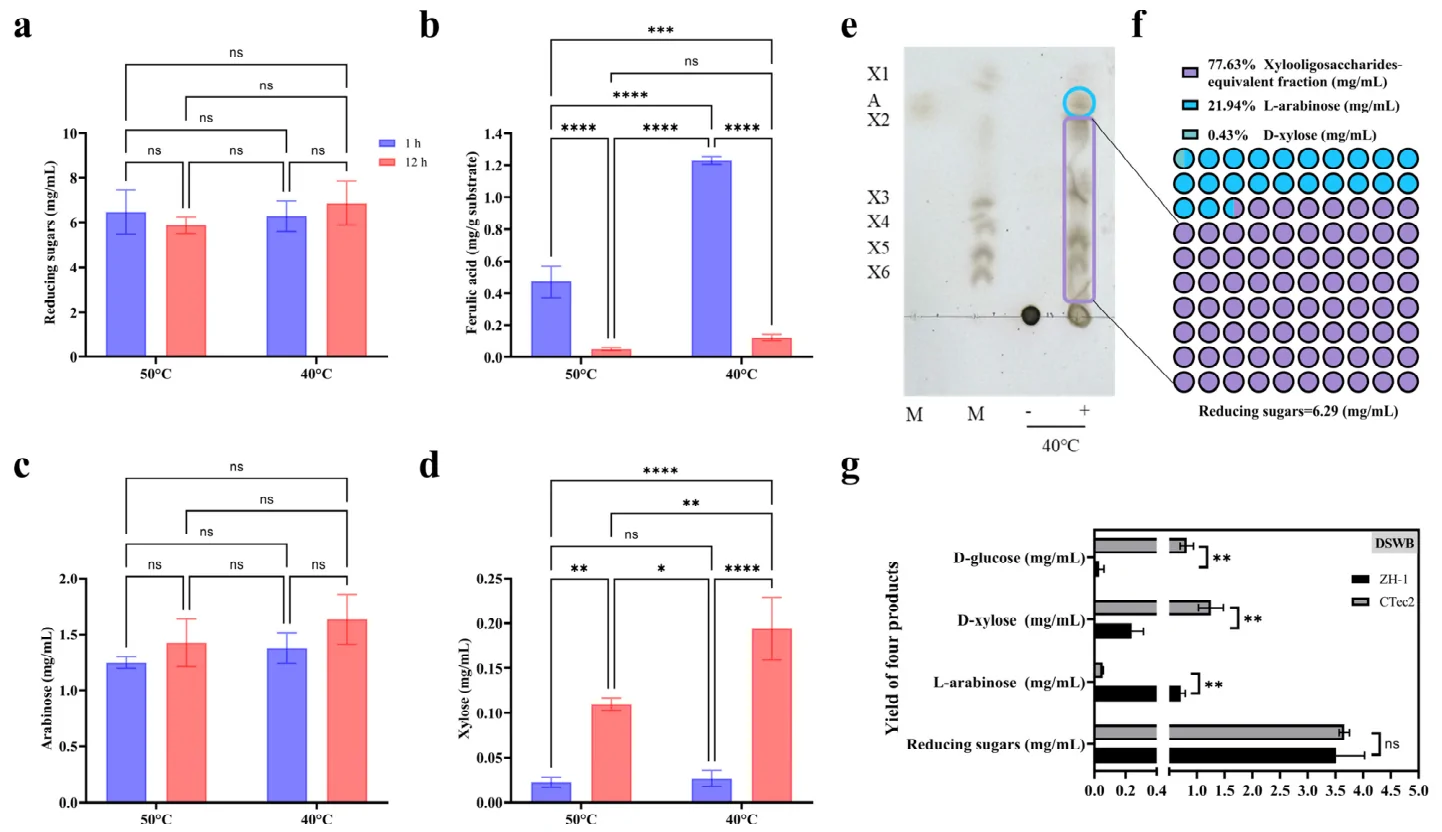
Hydrolysis products of ZH-1 enzyme cocktail and commercial CTec2 against insoluble wheat arabinoxylan (I-WAX) or de-starched wheat bran (DSWB). (**a**) Production of reducing sugars from I-WAX by ZH-1 enzyme cocktail. (**b**) Production of ferulic acid from I-WAX by ZH-1 enzyme cocktail. (**c**) Production of L-arabinose from I-WAX by ZH-1 enzyme cocktail. (**d**) Production of D-xylose from I-WAX by ZH-1 enzyme cocktail. (**e**) Thin-layer chromatography (TLC) patterns of I-WAX hydrolysates at 40 °C for 1 h by ZH-1 enzyme cocktail. M: mixture of xylose (X1), xylobiose (X2), xylotriose (X3), xylotetraose (X4), xylopentose (X5), xylohexose (X6), and arabinose (A); -: control, +: hydrolysates by ZH-1. (**f**) Proportions of arabinose, xylose, and xylooligosaccharides-equivalent fraction in the total reducing sugars of I-WAX hydrolysates at 40 °C for 1 h by ZH-1 enzyme cocktail. The xylooligosaccharides-equivalent fraction was determined by difference, i.e., total reducing sugars minus monosaccharides. (**g**) Comparison of hydrolysis products (reducing sugars, L-arabinose, D-xylose, and D-glucose) released from DSWB by ZH-1 enzyme cocktail and commercial CTec2 at 40 °C for 48 h. Data are presented as mean ± SD (*n* = 3). Statistical significance is indicated by ns (*p* ≥ 0.05), * (*p* < 0.05), ** (*p* < 0.01), *** (*p* < 0.001), and **** (*p* < 0.0001). For (**a**–**d**), statistical significance was determined by two-way ANOVA followed by Tukey’s post hoc test. For (**g**), an unpaired two-tailed *t*-test with Welch’s correction was applied.

Insoluble wheat arabinoxylan (I-WAX) was used as a model substrate to evaluate the application potential of the ZH-1 enzyme cocktail. At 40 °C and 1 h, the enzyme cocktail released 6.29 mg/mL (corresponding to 314.50 mg/g substrate) of reducing sugars from I-WAX, along with 1.38 mg/mL L-arabinose (corresponding to 69.00 mg/g substrate), 0.03 mg/mL D-xylose (corresponding to 1.50 mg/g substrate), and 1.23 mg/g substrate of ferulic acid (FA) (Figure 8a–d). Temperature and reaction time had limited effects on total reducing sugars and L-arabinose production, with no significant differences among all treatment groups (*p* > 0.05) (Figure 8a,c). However, temperature and reaction time significantly influenced FA and xylose release (Figure 8b,d). At 40 °C, the FA yield after 1 h of hydrolysis (1.23 mg/g I-WAX) was 2.62-fold higher than that at 50 °C (0.47 mg/g I-WAX). Likewise, at 12 h, the yield at 40 °C (0.12 mg/g I-WAX) was 2.40-fold higher than that at 50 °C (0.05 mg/g I-WAX) (Figure 8b). This temperature-dependent decrease in product yield is consistent with the findings of Ketsakhon et al. [46], who reported that the released reducing sugars from rice straw by the enzyme cocktail of *B. altitudinis* RS3025 decreased significantly with increasing reaction temperature. Notably, FA yield at 12 h decreased significantly regardless of temperature, a trend also observed by Sharma et al. [39]. The precise mechanism underlying this decrease remains unclear and may involve enzymatic conversion, adsorption onto residual substrate, or oxidative reactions; further studies with appropriate controls are needed to clarify this phenomenon.

TLC analysis (Figure 8e) confirmed the presence of L-arabinose and xylooligosaccharides (XOS) with a degree of polymerization ≥ 2 in the hydrolysates of I-WAX. The proportions of each component in total reducing sugars at 40 °C for 1 h were calculated (Figure 8f), with the xylooligosaccharides-equivalent fraction accounting for the highest proportion (approximately 77.63%), followed by L-arabinose (approximately 21.94%), and xylose at extremely low levels. These results are consistent with the substrate specificity assays (Section 3.4), which demonstrated that the enzyme cocktail preferentially acts on arabinoxylan-type substrates. It should be noted that this fraction was determined by difference (total reducing sugars minus monosaccharides) and therefore represents an equivalent fraction rather than directly quantified XOS. The high proportion of this xylooligosaccharides-equivalent fraction suggests potential food-related application prospects. Nevertheless, direct quantification of XOS would be needed to confirm whether it meets the commercial purity range (75–95%) typically required for food-grade XOS products [46]. Collectively, these results demonstrate that the major products (99.57%) generated by the ZH-1 enzyme cocktail from I-WAX are high-value functional saccharides (an xylooligosaccharides-equivalent fraction and the low-calorie sweetener L-arabinose), along with the natural antioxidant FA, highlighting the promising potential of this enzyme cocktail for the valorization of agricultural residues. It should be noted that the ferulic acid reported in this study was released from arabinoxylan-rich substrates through in vitro enzymatic hydrolysis by the ZH-1 enzyme cocktail, rather than being generated by the strain itself.

Building on the results obtained with the model substrate, de-starched wheat bran (DSWB) was further used as a natural substrate to validate the hydrolytic performance of the enzyme cocktail on real agricultural residues, and the hydrolysis performance was compared with that of the commercial cellulase Cellic^®^ CTec2. CTec2 is a commercial cellulase preparation produced by *Trichoderma reesei*, containing at least two main cellobiohydrolases, five different endo-1,4-β-glucanases, β-glucosidase, β-xylosidase, and GH61 proteins [47]. This enzyme cocktail has also been reported to exhibit high tolerance to product inhibition [48] and is optimized for the saccharification of lignocellulosic biomass, particularly corn stover [49]. Therefore, CTec2 was selected as a reference to evaluate the relative performance of the ZH-1 enzyme cocktail. Given that no significant difference in total reducing sugar yield was observed between 40 °C and 50 °C during I-WAX hydrolysis (Figure 8a), and considering energy consumption and potential enzyme inactivation during prolonged incubation (48 h), DSWB hydrolysis was conducted at 40 °C (Figure 8g). The total reducing sugar yields of ZH-1 and CTec2 showed no significant difference (3.51 vs. 3.66 mg/mL; *p* > 0.05), corresponding to 175.50 vs. 183.00 mg/g substrate, respectively. However, their monosaccharide composition differed markedly (*p* < 0.05). ZH-1 preferentially released L-arabinose (0.71 mg/mL, corresponding to 35.5 mg/g substrate), while CTec2 predominantly produced fermentable sugars, including glucose (0.82 mg/mL) and xylose (1.25 mg/mL). In contrast, ZH-1 released only trace amounts of glucose and xylose (0.03 and 0.24 mg/mL, respectively). Calculation of the monosaccharide proportion in total reducing sugars revealed that CTec2 exhibited a fermentable sugar ratio of 58.04%, while ZH-1 showed only 27.91%, with 72.40% of the monosaccharide pool consisting of the low-calorie sweetener L-arabinose. This indicates that the ZH-1 hydrolysate still contained a considerable proportion of oligosaccharides (mainly XOS), which can be attributed to the absence of β-xylosidase in the enzyme cocktail (Section 3.4). These comparative results suggest that different enzyme cocktails can be selected for targeted conversion depending on the desired products. The lack of detectable β-xylosidase activity in the ZH-1 cocktail prevents the further hydrolysis of XOS into xylose, thereby likely contributing to the accumulation of oligosaccharides. This is particularly advantageous over commercial cellulase preparations (e.g., CTec2), which contain β-xylosidase and thus shift the product spectrum toward fermentable monosaccharides rather than functional oligosaccharides. Additionally, no free ferulic acid was detected in the DSWB hydrolysate. The reason for the absence of free FA remains to be determined; it may involve enzymatic conversion, adsorption, or other processes, and requires further investigation. The hydrolytic performance of ZH-1 is also comparable to or better than that of other *B. altitudinis* strains previously reported. For instance, the enzyme cocktail of *B. altitudinis* RS3025 produced 146.87 mg/g reducing sugars from alkaline-treated rice straw at 30 °C for 24 h [45], while that of *B. altitudinis* X-10 yielded 14.22 mg/g from 100-mesh wheat straw at 50 °C for 30 min [31]. Although direct comparison of lignocellulose conversion efficiency across different studies is challenging due to variations in substrates and conditions, these results demonstrate that the ZH-1 enzyme cocktail is a promising candidate for the high-value valorization of arabinoxylan-rich agricultural residues.

From a practical perspective, the ZH-1 enzyme cocktail exhibits several advantages for biorefinery applications. Its ability to produce a xylooligosaccharides-equivalent fraction and L-arabinose, both of which represent promising food-relevant functional ingredients, supports its use in the production of value-added food and nutraceutical products. In addition, the absence of detectable β-xylosidase activity under the assay conditions prevents excessive hydrolysis of XOS into xylose, allowing better control over the degree of polymerization of the final oligosaccharide products. Compared with commercial cellulase preparations such as CTec2, which favor the release of fermentable monosaccharides, the ZH-1 cocktail provides a complementary product profile enriched in functional oligosaccharides. Combined with the use of low-cost substrates such as wheat bran, these features highlight the potential of the ZH-1 enzyme system for sustainable and cost-effective production of functional sugars from agricultural by-products. Nevertheless, the present findings are derived from laboratory-scale batch hydrolysis assays; further work concerning process optimization and techno-economic evaluation will be required to support its real-world implementation.

## 4. Conclusions

This work demonstrates that *Bacillus altitudinis* ZH-1 is a valuable feruloyl esterase-producing strain. Genomic analysis and enzymatic profiling collectively show that its extracellular enzyme cocktail is dominated by hemicellulases and shows no detectable β-xylosidase activity under the assay conditions. This feature gives it a distinct advantage for arabinoxylan bioconversion: it favors the accumulation of a xylooligosaccharides-equivalent fraction instead of further hydrolyzing them into xylose. Hydrolysis assays further confirm its capacity to generate functional sugars from lignocellulosic feedstocks, and its performance is comparable to that of a commercial enzyme preparation, CTec2, while favoring the accumulation of target functional sugars. Collectively, our findings indicate that strain ZH-1 represents an attractive resource for valorizing arabinoxylan-rich agricultural residues in food-related and nutraceutical applications. Further research can be directed toward enzyme cocktail optimization and scale-up studies for industrial applications.

## Figures and Tables

**Table 1:** Comparison of feruloyl esterase production by different microbial strains under submerged fermentation conditions.

	Strain	Source	Fermentation Conditions	Maximum Feruloyl Esterase (FAE) Activity (U/L)	Reference
Bacteria	*Bacillus altitudinis* ZH-1	Distiller’s grain	34 °C; 0 rpm; 72 h; pH = 7.5	1194.79	This study
	*Bacillus altitudinis* ZH-1	Distiller’s grain	34 °C; 0 rpm; 72 h; pH = 7.5	1015.57 ^a^	This study
	*Bacillus pumilus* SK52.001	Soil	30 °C; 200 rpm; 26 h; pH = -	195	[13]
	*Lactiplantibacillus pentosus*	Silages	37 °C; - rpm; - h; pH = -	7.11	[14]
	*Acetobacter* sp. J1	Jiupei	30 °C; 180 rpm; 24 h; pH = 7.0	3.83 ^a^	[20]
	*Klebsiella oxytoca* Z28	Daqu	37 °C; 180 rpm; 48 h; pH = -	35 ^a^	[21]
	*Enterococcus lactis* SR1	Sauerkraut	37 °C; 0 rpm; 72 h; pH = 6.5	7.09 ^a,b^	[39]
Actinomycetes	*Nonomuraea* sp. ID06-094	Indonesian Institute of Sciences	28 °C; 190 rpm; 168 h; pH = -	4.0	[17]
	*Streptomyces* S_10_	Soil	30 °C; - rpm; 96 h; pH = 7.0	2.0	[40]
Fungi	*Aspergillus terreus*	Soil	35 °C; 150 rpm; 120 h; pH = 6.0	6700	[23]
	*Humicola grisea* var. *thermoidea*	-	37 °C; 150 rpm; 192 h; pH = -	470	[37]
	*Penicillium brasilianum* IBT 20888	-	30 °C; 200 rpm; 120 h; pH = 5.0	225	[38]
	*Hericium erinaceus*	Purchased	25 °C; 100 rpm; 96 h; pH = 6.0	2.04	[41]
	*Penicillium decumbens*	Purchased	30 °C; 200 rpm; 144 h; pH = 5.0	3.22 ^a^	[42]

^a^: Activities of the cited references were determined using ethyl ferulate as the substrate, whereas methyl ferulate was used in others. Two separate entries for *Bacillus altitudinis* ZH-1 are presented, one assayed with methyl ferulate (MFA) and one with ethyl ferulate (EFA), to allow comparison with the literature data determined with different substrates. ^b^: The enzyme unit definition in Sharma et al. [39] was based on the release of 1 µM ferulic acid in a 1 mL assay system (1 IU = 1 nmol/min), whereas 1 U in this and other studies corresponds to 1 µmol/min. For comparison, values from Sharma et al. [39] were converted to U/L accordingly (1 IU/mL = 1 U/L in this context). -: not reported in the original reference.

## Data Availability

The original contributions presented in this study are included in the article/Supplementary Materials. Further inquiries can be directed to the corresponding author. The whole-genome shotgun project of *Bacillus altitudinis* ZH-1 has been deposited in GenBank under accession number JBZBUO000000000 (BioProject: PRJNA1476822; BioSample: SAMN60733002). The version described in this paper is JBZBUO010000000. The genome assembly and its associated metadata are now publicly available.

## Supplementary Materials

The following supporting information can be downloaded at: https://www.mdpi.com/article/10.3390/foods15203642/s1, Figure S1: Graphical circular genome map of *B. altitudinis* ZH-1; Table S1: Summary of reported feruloyl esterase-producing strains isolated from traditional Chinese fermentation systems. Table S2: Preliminary screening of different medium types for feruloyl esterase production by *Bacillus altitudinis* ZH-1. Table S3: Results of dDDH and ANI analyses for *B. altitudinis* ZH-1. Table S4: The basic genome features of *B. altitudinis* ZH-1.; Table S5: Comparative genomic analysis of ZH-1’s CAZymes with other *B. altitudinis* strains.Table S6: Annotation of key CAZymes from *B. altitudinis* ZH-1 based on sequence homology and structural templates.
