# Supplementary material for: A Novel Feruloyl Esterase-Producing Strain ZH-1 Belonging to Bacillus altitudinis from Baijiu Distiller’s Grains and Its Enzyme Cocktail with No Detectable β-Xylosidase Activity for Producing Functional Sugars from Arabinoxylan-Type Substrates
Source: Foods. 2026 Oct 14;15(20):3642. doi: 10.3390/foods15203642 (PMC13704613; doi:10.3390/foods15203642)
Supplement: Supplementary file 1 [file foods-15-03642-s001.zip › foods-4577674-supplementary.pdf]

Figure S1 Graphical circular genome map of *B. altitudinis* ZH-1.

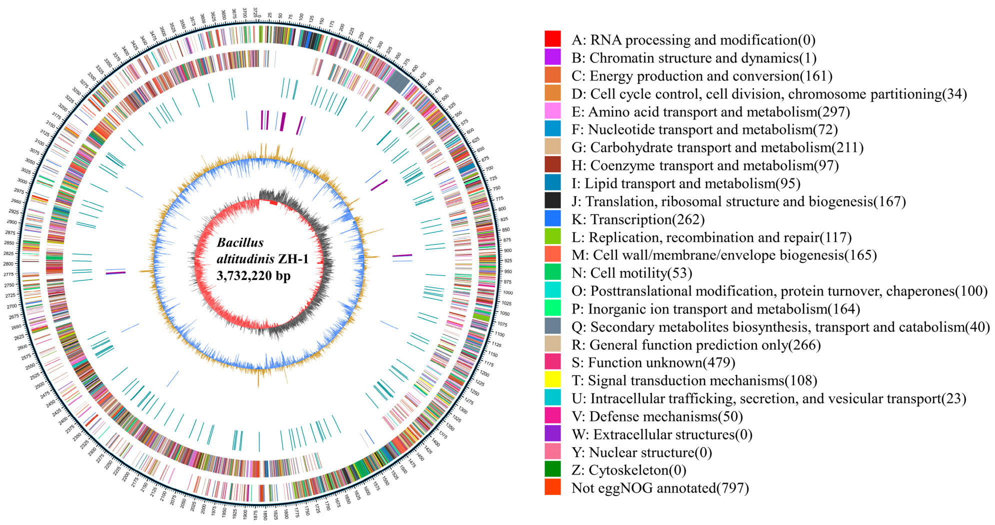

Table S1 Summary of reported feruloyl esterase-producing strains isolated from traditional Chinese fermentation systems.

Strain	Source	Fermentation mode	FAE-related parameter	Reference
Acetobacter sp. J1	Jiupei	Submerged fermentation	3.83 U/L	[20]
Klebsiella oxytoca Z28	Daqu	Submerged fermentation	35 U/L	[21]
Penicillium oxalicum M1816	Huangjiu wheat Qu	Solid-state fermentation	1.85 mg ferulic acid/g substrate	[22]

J1 and Z28 were cultivated under submerged fermentation, and their FAE activities are expressed in U/L. M1816 was cultivated under solid-state fermentation, and its FAE-related parameter represents ferulic acid-releasing yield (1.85 mg ferulic acid per g substrate). Due to the different fermentation modes, these values are not directly comparable.

Table S2 Preliminary screening of different medium types for feruloyl esterase production by *Bacillus altitudinis* ZH-1.

**Medium formulation**	**FAE activity (U/L)**
LB (unoptimized control, yeast extract 5 g/L, peptone 10 g/L, NaCl 10 g/L; initial pH 7.0)	75.70 ± 5.04
Medium 1 (yeast extract 5 g/L, peptone 10 g/L, NaCl 10 g/L, ethyl ferulate solution (10% w/v in N,N-dimethylformamide) 4 mL/L; initial pH 7.0)	98.40 ± 2.05
Medium 2 (KH_2PO_4 0.6 g/L, $(NH_4)_2SO_4$ 1.4 g/L, $MgSO_4·7H_2O$ 0.48 g/L, $CaCl_2·2H_2O$ 0.086 g/L, $FeCl_3$ 0.03 g/L, yeast extract 2.29 g/L, ethyl ferulate solution (10% w/v in N,N-dimethylformamide) 8 mL/L; initial pH 5.5)	not detected
Medium 3 (yeast extract 1 g/L, glucose 6 g/L, peptone 1 g/L, $CaCO_3$ 1 g/L, $MgSO_4·7H_2O$ 0.05 g/L, $NaH_2PO_4·2H_2O$ 2 g/L, $Na_2HPO_4·2H_2O$ 4 g/L; initial pH 7.2)	not detected
Selected production medium (glucose 30 g/L, yeast extract 60 g/L, wheat bran 30 g/L, KCl 0.05 g/L, $MgSO_4·7H_2O$ 0.05 g/L, K_2HPO_4 0.10 g/L; initial pH 6.5)	244.92 ± 12.18

All fermentations were performed under identical initial culture conditions (37 °C, 150 rpm, 72 h). FAE activity values represent mean ± standard deviation of three biological replicates.

Table S3 Results of dDDH and ANI analyses for *B. altitudinis* ZH-1.

**Strain**	**Reference strain**	**Assembly accession**	**dDDH value (d_0, %)**	**OrthoANIu value (%)**	**GC difference (%)**
ZH-1	*Bacillus altitudinis* 41KF2b^T (= DSM 21631^T)	GCA_000691145	95.40	98.50	0.12
ZH-1	*Bacillus pumilus* ATCC 7061^T	GCA_000172815	85.80	88.95	0.28
ZH-1	*Bacillus safensis* subsp. *osmophilus* CECT 9344^T (= BC 9^T)	GCA_003254445	82.50	89.04	0.50

dDDH, digital DNA-DNA hybridization; OrthoANIu, average nucleotide identity calculated using usearch. dDDH values were computed using formula d_0 within the TYGS-GGDC 4.0 workflow. Repeated processing errors occurred during attempts to compute average amino-acid identity (AAI) with public web-servers; hence, AAI results are not included in this table.

Table S4 The basic genome features of *B. altitudinis* ZH-1.

Features	Results	Features	Results
Genome size (bp)	3,732,220	23S rRNA	8
G+C content	41.39%	NR annotation	3,744
Coding genes	3,759	eggNOG annotation	2,962
Average length (bp)	872	GO annotation	2,932
Coding genes/Genome (%)	87.86	KEGG annotation	2,723
tRNA	81	Swiss-Prot annotation	3,126
5S rRNA	8	Pfam annotation	3,316
16S rRNA	8	TrEMBL annotation	3,740

Table S5 Comparative genomic analysis of ZH-1's CAZymes with other *B. altitudinis* strains.

***B. altitudinis* strains**	**GHs**	**CEs**	**GTs**	**AAs**	**PLs**	**CBMs**	**Total domains** [a]	**Total genes** [b]
ZH-1	38	23	35	3	2	5	106	103
SRL571	35	21	36	4	3	5	104	101
JYY-02	39	22	36	4	2	5	108	105
B12	40	22	33	4	2	5	103	100
W3	39	22	34	4	2	5	103	100

a: Total domains represent the cumulative count of all CAZyme domains identified (GHs + CEs + GTs + AAs + PLs + CBMs).

b: Total genes represent the number of unique genes encoding at least one CAZyme domain after removing redundant counts caused by multi-domain proteins.

Table S6 Annotation of key CAZymes from *B. altitudinis* ZH-1 based on sequence homology and structural templates.

Classification	PDB-ID	Gene-ID	CAZy family	Type of enzyme activity	Similarity	Signal peptide
Cellulose-related	8U49	GE001592	GH9-CBM3	Endo-β-1,4-glucanase	67.70%	No
	7D88	GE001594	GH10	Endo-β-1,4-glucanase/Endo-β-1,4-xylanase	97.55%	No
	5BV9	GE001593	GH48	Cellobiohydrolase	92.30%	No
	1GBG	GE000498	GH16_21	Endo-β-1,3-1,4-D-glucanase	98.60%	Yes (1-29)
Hemicellulose-related	7D88	GE001594	GH10	Endo-β-1,4-glucanase/Endo-β-1,4-xylanase	97.55%	No
	1IGO	GE001861	GH11	Endo-β-1,4-xylanase	84.58%	Yes (1-28)
	3GTN	GE001775	GH30_8	Glucuronoxylanase	85.64%	Yes (1-34)
	5ZQJ	GE001865	GH43_11-CBM91	β-xylosidase	99.25%	No
	3C7G	GE001776	GH43_16-CBM6	Arabinoxylan arabinofuranohydrolase	90.35%	Yes (1-29)
	2VRQ	GE002553	GH51_1	α-L-Arabinofuranosidase	61.48%	No
	8GF3	GE001595	GH5_7	Endo-β-1,4-mannanase	73.64%	Yes (1-29)
	6RZO	GE003725	CE1	Putative feruloyl esterase	21.62%	No
	7XRT	GE001141	CE1	Putative feruloyl esterase	22.69%	No
	8JH8	GE003466	CE1	Putative feruloyl esterase	25.22%	Yes (1-30)
	7Y51	GE002343	CE4	Acetyl xylan esterase	39.15%	No
	1ZMB	GE001147	CE6	Acetylxylan esterase	60.00%	No
	1ZMB	GE001152	CE6	Acetylxylan esterase	61.45%	No
	2XLB	GE000330	CE7	Acetyl xylan esterase	99.43%	No
Other	7BKF	GE001607	CE4	Polysaccharide deacetylase	56.25%	Yes (1-23)
	6DQ3	GE003553	CE4	Polysaccharide deacetylase	40.18%	Yes (1-27)
	7QJM	GE002711	CE19	alpha/beta-hydrolase	36.04%	No

The three CE1 genes (GE003725, GE001141, and GE003466) were designated as putative feruloyl esterase-encoding genes based on low sequence identities (21.62-25.22%), and their actual FAE activity requires further biochemical validation.
